# Tenofovir Inhibits Wound Healing of Epithelial Cells and Fibroblasts from the Upper and Lower Human Female Reproductive Tract

**DOI:** 10.1038/srep45725

**Published:** 2017-04-03

**Authors:** Marta Rodriguez-Garcia, Mickey V. Patel, Zheng Shen, Jack Bodwell, Richard M. Rossoll, Charles R. Wira

**Affiliations:** 1 Department of Microbiology and Immunology, Geisel School of Medicine at Dartmouth, One Medical Center Drive, Lebanon, NH, 03756 USA

## Abstract

Disruption of the epithelium in the female reproductive tract (FRT) is hypothesized to increase HIV infection risk by interfering with barrier protection and facilitating HIV-target cell recruitment. Here we determined whether Tenofovir (TFV), used vaginally in HIV prevention trials, and Tenofovir alafenamide (TAF), an improved prodrug of TFV, interfere with wound healing in the human FRT. TFV treatment of primary epithelial cells and fibroblasts from the endometrium (EM), endocervix (CX) and ectocervix (ECX) significantly delayed wound closure. Reestablishment of tight junctions was compromised in EM and CX epithelial cells even after wound closure occurred. In contrast, TAF had no inhibitory effect on wound closure or tight junction formation following injury. TAF accumulated inside genital epithelial cells as TFV-DP, the active drug form. At elevated levels of TAF treatment to match TFV intracellular TFV-DP concentrations, both equally impaired barrier function, while wound closure was more sensitive to TFV. Furthermore, TFV but not TAF increased elafin and MIP3a secretion following injury, molecules known to be chemotactic for HIV-target cells. Our results highlight the need of evaluating antiretroviral effects on genital wound healing in future clinical trials. A possible link between delayed wound healing and increased risk of HIV acquisition deserves further investigation.

The global HIV pandemic is now in its third decade with 36.7 million living with HIV in 2015^1^. On a worldwide basis, young women aged 15**–**24 are most vulnerable to HIV infection, with sexual intercourse the predominant mechanism for HIV transmission. Young women are particularly vulnerable to HIV infection, with rates 2-fold higher than young men on a worldwide basis and 8-fold higher in Sub-Saharan Africa^2^.

Overlapping the prevalence of HIV infection worldwide is the pandemic of sexual violence against women. In the USA and Africa, 23–37% of girls and women experience sexual abuse with 80% of victims younger than 30 years of age^3^. Not widely appreciated is the high frequency of genital injury that occurs with sexual intercourse^4^. In a recent study, 55% of women examined following consensual intercourse had at least 1 anal-genital injury^5^, with 50% of women showing internal genital injuries. A recent meta-analysis of 16 countries showed that Intimate Partner Violence, frequently associated with genital violence, is significantly associated with HIV infection^6^. In addition to injuries in the lower tract due to sexual intercourse, different causes can induce epithelial disruption in the upper tract, including cervical ectopy^7^, menstruation or ascending infections. While it is generally assumed that HIV reaches target cells through breaches in the epithelium, little is known about the factors that influence the repair process of the mucosal barrier following disruption.

Topical or oral administration of antiretrovirals (ARVs) is one of the current interventions under investigation for preventing HIV infection in women. Oral administration of tenofovir disoproxil fumarate in combination with emtricitabine is recommended for use as pre-exposure prophylaxis (PrEP) in people at high risk for HIV acquisition^8^, after efficacy was demonstrated in several trials^9^,^10^,^11^. However, clinical trials involving only women have been ineffective in preventing HIV acquisition, and showed lower concentrations of TFV in plasma^12^,^13^. Of three trials conducted in Africa to test the efficacy of topical TFV (CAPRISA 004, FACTS and VOICE), only CAPRISA 004 showed significant but moderate protection against HIV acquisition^12^,^14^. While compliance is a major factor accounting for these mixed results^15^, the contribution of other biological factors remains to be investigated.

Recently, a new pro-drug of TFV, Tenofovir alafenamide (TAF; formerly known as GS-7340), with increased efficacy and reduced toxicity was approved for HIV treatment^16^. In addition, the recognition that TAF preferentially targets lymphoid tissues and HIV susceptible cells when compared to TFV^17^,^18^, raises the likelihood that it will be part of the next generation of ARVs used in the global prevention of HIV^19^.

Inside the cells, TFV and TAF are enzymatically converted to TFV diphosphate (TFV-DP), which is the active drug form with anti-HIV activity, with TAF reaching higher intracellular levels than TFV^16^. We have previously demonstrated that TFV treatment of FRT cells results in TFV-DP accumulation in epithelial cells and fibroblasts throughout the FRT, with concentrations several fold higher than immune cells^20^. In addition, we demonstrated that TFV stimulates the secretion of proinflammatory cytokines by epithelial cells, fibroblasts and immune cells from the FRT^21^. However, effects of TAF on primary genital epithelial cells have not been evaluated.

To date, genital trauma (present after consensual and non-consensual sex) has not been considered as a confounding factor in the success or failure of microbicide clinical trials designed to prevent HIV acquisition. Since TFV clearly accumulates in and stimulates the secretion of cytokines and chemokines by epithelial cells and fibroblasts from the FRT^20^,^21^, here we investigate the possible effects of TFV and the new pro-drug TAF on wound healing in the human FRT. Using an *in vitro* scratch wound model, we report that at clinically relevant concentrations TFV, but not TAF, suppress wound healing of primary FRT epithelial cells and fibroblasts. This discovery suggests that beyond their anti-HIV effects, some ARVs may compromise wound healing, a key determinant in immune protection, thereby extending the time of tissue immune cell exposure to HIV. Our findings highlight the importance of evaluating ARV interference with wound healing for future clinical trials to identify the right ARVs and doses that will not compromise mucosal immune protection or interfere with wound healing while maintaining anti-HIV activity.

## Methods and Materials

### Study Subjects

Reproductive tract tissues from the endometrium (EM), endocervix (CX) and ectocervix (ECX) were obtained from 28 women undergoing hysterectomy surgery at Dartmouth-Hitchcock Medical Center (Lebanon, NH). Women were aged between 28 and 58 years old. All tissues used in this study were distal from the sites of pathology and were determined to be unaffected with disease upon inspection by a pathologist. All investigations were conducted according to the principles expressed in the Declaration of Helsinki and carried out with the approval from the Committee for the Protection of Human Subjects (CPHS), Dartmouth Hitchcock Medical Center, and with written informed consent obtained from the patients before surgery.

### Isolation of Epithelial Cells and Fibroblasts

Tissues were minced under sterile conditions into 1 to 2 mm fragments and enzymatically digested using a mixture consisting of 0.05% collagenase type IV (Sigma-Aldrich, St Louis, MO) and 0.01% DNAse (Worthington Biochemical, Lakewood, NJ) for 1 hr at 37 °C as described before^20^. After enzymatic digestion, cells were dispersed through a 250 μm mesh screen (Small Parts, Miami Lakes, FL), washed, and resuspended in Hank’s Balanced Salt Solution (HBSS) (Thermo Fisher, Logan, UT).

Epithelial cell sheets (containing glands and luminal cells in variable proportions) were separated from stromal fibroblasts by filtration through a 20 μm nylon mesh filter (Small Parts) as previously described^20^,^21^,^22^. Epithelial sheets were retained on the 20 μm filter, while the stromal fraction containing the fibroblasts, passed through and collected as part of the filtrate. Epithelial sheets were recovered by rinsing and backwashing the filter with DMEM/F12 (Thermo Fisher), centrifuged (500 x *g,* 10 min), and analyzed for cell number and viability.

### Epithelial cell culture

Isolated sheets of EM, CX and ECX epithelial cells were plated in 0.4 μm 24-well plate transwell inserts (Corning Life Sciences, Tewksbury, MA) and grown in defined media consisting of DMEM/F12 (Thermo Fisher) supplemented with NuSerum (Corning Life Sciences), Hyclone Defined FBS (GE Life Sciences, Logan, UT), Penicillin-Streptomycin (Thermo Fisher), L-Glutamine (Thermo Fisher) and HEPES (GE Life Sciences). EM and CX epithelial cells were grown to confluence and allowed to polarize as determined by transepithelial resistance (TER) of greater than 1000 ohms/cm^2^ per insert. TER was measured using an EVOM electrode and Voltohmmeter (World Precision Instruments, Sarasota, FL). Only polarized preparations of EM and CX epithelial cells were used in our studies. ECX epithelial cells, being squamous in phenotype, do not polarize and were grown to confluence on transwell inserts prior to treatment. 24 h before tenofovir (TFV) or tenofovir alafenamide (TAF) treatment, cells were transferred in to stripped media. Stripped media was DMEM/F12 supplemented with stripped FBS (Gemini Bio-Products, West Sacramento, CA), Penicillin-Streptomycin (Thermo Fisher), L-Glutamine (Thermo Fisher), and HEPES (GE Life Sciences).

### Stromal Fibroblast cell culture

Stromal cell suspensions from the EM, CX and ECX were cultured in T-75 flasks (Corning Life Sciences) in stromal defined media consisting of DMEM/F12 (Thermo Fisher) supplemented with Hyclone Defined FBS (GE Life Sciences), Penicillin-Streptomycin (Thermo Fisher), L-Glutamine (Thermo Fisher) and HEPES (GE Life Sciences). Once cells reached confluence they were passaged using trypsin-EDTA and grown to confluence again. After 2–3 passages, a purified population of stromal fibroblasts remained that is characterized as vimentin+, CD90+, CD45− and EpCam- (not shown).

### Tenofovir and Tenofovir Alafenamide treatment

Tenofovir (TFV) was obtained from the AIDS Research and Reference Reagent Program (NIH AIDS Reagent Program, Division of AIDS, NIAID, NIH: Tenofovir, catalog number 10199). TAF was kindly supplied by Gilead Sciences Inc. (Foster City, CA). A stock concentration of TFV (5 mg/ml) was prepared by adding 1 ml of PBS to 5 mg of TFV powder before being diluted in stripped media to the appropriate working concentration^20^,^21^. Tenofovir Alafenamide (TAF) was dissolved in PBS at 10 mM, sterilely filtered (0.2 μm) and the concentration checked by absorbance using a molar extinction coefficient of 11,690 at 260 nm. Subsequent dilutions were made in media to the final concentrations. TFV or TAF were added to epithelial cells or fibroblasts for 24 h prior to scratch and maintained in the culture media throughout each experiment. Untreated control cells were donor-matched to treated cells and scratch and assessment of wound healing was done in parallel. Cell viability was tested after treatment using the CellTiter 96 AQ_ueous_ One Solution cell proliferation assay (Promega, Madison, WI, USA) and trypan blue staining (HyClone Laboratories, Inc., Logan, UT) as described before^21^ and no changes in viability were found.

### Determination of intracellular TFV-DP levels

After 24 h treatment with TFV or TAF, epithelial cells were harvested and lysed in 300 μl of 70% methanol, and stored immediately at −80 °C prior to TFV-DP evaluation as previously described^20^,^23^. Intracellular TFV-DP concentrations were measured by liquid chromatography with tandem mass spectrometry (LC-MS/MS) and normalized for cell count.

### Scratch Assay

Confluent monolayers of epithelial cells in transwell inserts and stromal fibroblasts in 24-well plates were scratched using a 20 μl pipette tip attached to a wooden handle. This allows for scratches of consistent width and length. Scratch width and TER was measured immediately afterwards and at regular intervals as noted in the Results. Plates were marked immediately after scratch to ensure that scratches were measured at the same location throughout each experiment. Cell proliferation was determined using the CellTiter Aqueous One cell proliferation assay (Promega Corporation, Madison, WI) according to the manufacturer’s instructions.

### Wound healing visualization

To visualize the wound healing process, epithelial cells from the EM and the ECX were plated in 96-well plates and scratched as described above. Immediately after scratch, plates were inserted in the IncuCyte Zoom system (Essen Bioscience, Ann Arbor, MI) to acquire images. Serial images were taken every two hours for EM epithelial cells and every 15 min for ECX epithelial cells, for a 24–48 h period with a 10X objective. Images were compiled to generate movies.

### Measurement of acute responses

EM epithelial cells were treated with TFV or TAF for 24 h, washed and scratched and cell culture media collected 3 h following scratch. Five different molecules known to have anti-HIV activity were evaluated in culture supernatants using a custom microsphere multiplex assay described previously^24^,^25^. Briefly, commercially available antibody pairs were alternatively coupled to fluorescently-coated magnetic beads to capture analytes for elafin, HBD2, RANTES (CCL5), and MIP-3α (CCL20) and SDF-1 (CXCL12). ENA-78 (CXCL5) was measured using an ELISA assay kit (R&D, Minneapolis, MN USA) according to the manufacturer’s instructions.

### Statistics

Data analysis was performed using the GraphPad Prism 5.0 (GraphPad Software, La Jolla, CA). A two sided P value < 0.05 was considered statistically significant. Comparison of treatment groups vs. control group was performed applying Mann-Whitney U test for non-matched samples or Wilcoxon matched-pairs signed rank test for matched samples. Comparison of three or more groups was performed applying Kruskal-Wallis test for non-matched samples or Friedman test for matched samples, followed by Dunns-post test for multiple comparison correction. Dose-response time-course studies were analyzed using two-way ANOVA with Bonferroni post-test for multiple comparison correction.

## Results

### *In vitro* scratch assay model for wound healing in the FRT

**Figure 1:**
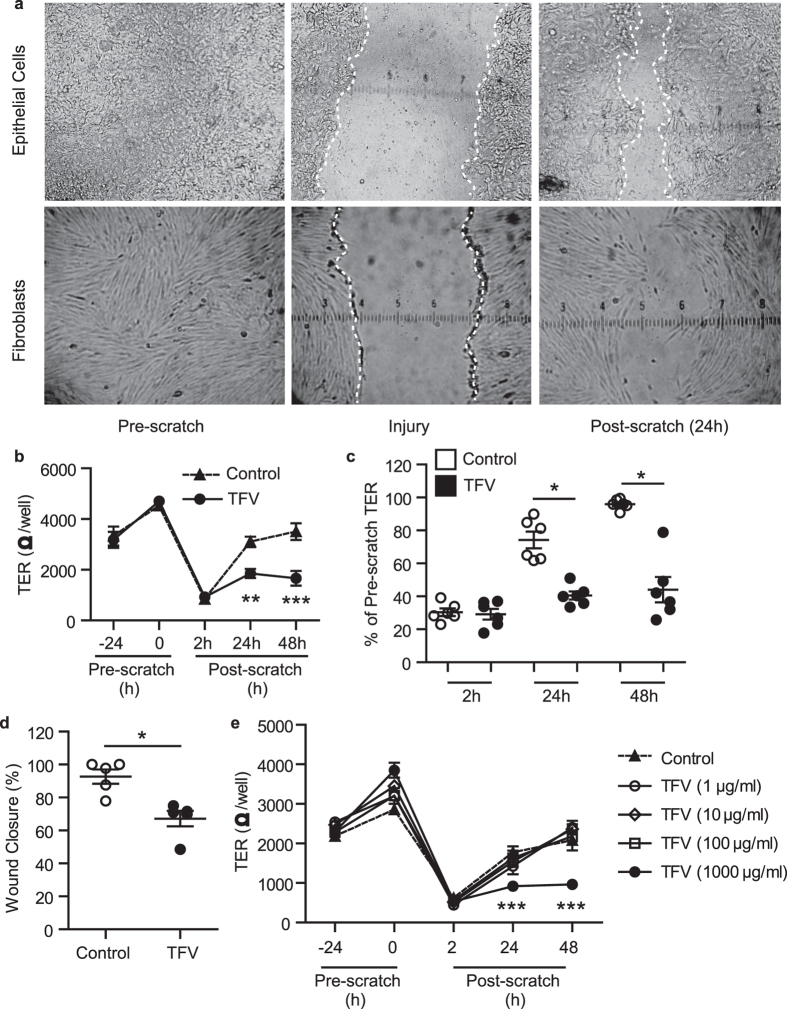
The effect of TFV on wound healing of uterine epithelial cells and fibroblasts in culture. (**a**) Sample images of *in vitro* wound healing model. Epithelial cells (top left panel) or fibroblasts (bottom left) were grown to confluence on inserts or 24 well plates respectively, as detailed in Methods. Cells were scratched (middle top and bottom panels, white lines indicate the margin of the wound) and wound closure measured. Right top and bottom panels show partial healing that takes place after 24 h. (**b**) Time course of the effect of TFV inhibition of uterine epithelial cells TER and wound closure following scratch. TER and scratch width measurements of polarized cultures of EM epithelial cells incubated with TFV (1 mg/ml; 3277 μM) 24 h before scratch and 2 h, 24 h and 48 h after scratch. Representative example of TER values (**b**) and normalized data to % of Pre-scratch TER values (**c**) in experiments using epithelial cells from 6 patients at 24 h and 48 h after scratch. (**d**) % of wound closure for epithelial cells from 5 patients at 24 h post scratch. Each circle represents a different patient. The mean and SEM are shown. *p < 0.05. (**e**) Dose response of the effect of TFV on inhibition of uterine epithelial cell TER. TER was measured for polarized EM epithelial cells incubated with increasing doses of TFV (1 μg/ml up to 1 mg/ml) for 24 h before scratch and 2 h, 24 h and 48 h following scratch. One representative example of three independent experiments with 3 different donors. The mean and SEM are shown from triplicate cultures of cells from a representative patient. ***p < 0.001.

To examine the mechanisms involved in trauma-induced wound repair of the FRT mucosal barrier, which is essential for re-establishing homeostasis and suppressing mucosal inflammation, we developed an *in vitro* system using primary epithelial cells and stromal fibroblasts from the upper (endometrium (EM) and endocervix (CX)) and lower FRT (ectocervix (ECX)). As seen in Fig. 1a, EM epithelial cells were grown to confluence on transwell inserts until they polarized and formed tight junctions, as detailed in Methods (1a, top left). Epithelial cell monolayers were uniformly scratched using a pipette tip applicator. The width of the wound was determined by tip size (Top middle panel: wound area is between the white lines). This approach was used to culture cells from CX and ECX (not shown). Primary fibroblasts were grown to confluence in 24 well plates (1a, bottom left) and scratched as described above (Bottom middle panel). Partial healing takes place by 24 h after scratch for epithelial cells and fibroblasts (top and bottom right panels respectively). Supplementary movie 1 shows wound closure of EM epithelial cells for 48 h following scratch.

### High doses of TFV inhibit wound healing by epithelial cells in the FRT

Recognizing that high doses of TFV are applied topically into the vagina in clinical trials to prevent HIV infection^14^, we asked whether TFV might influence barrier function and wound healing in the FRT. Using TFV at a concentration comparable to vaginal concentrations used in topical microbicide trials^14^, we incubated polarized EM epithelial cells with TFV (1 mg/ml; 3277 μM) for 24 h prior to injury. As seen in Fig. 1b, pretreatment with TFV had no effect on epithelial integrity measured as TER in the 24 h prior to scratch. However, following mechanical injury, TFV treatment blocked the re-establishment of tight junctions at 24 and 48 h, while traumatized-untreated control cells returned to pre-scratch TER levels by 48 h. Analysis of epithelial cells from different women (N = 6) demonstrated that TER recovery after scratch of TFV-treated cells was significantly decreased compared to untreated controls both at 24 and 48 h (Fig. 1c). TFV treatment also delayed wound closure, measured as the width of each scratch after 24 h (Supplementary movies 2 and 3). In all cases (Fig. 1d), closure was incomplete at 24 h (N = 5) but most often complete by 48 h (not shown). These findings indicate that TFV delays both wound closure and the re-establishment of barrier function measured as TER.

Recognizing that the concentrations of TFV that reach the upper tract may be lower than those found in the lower tract, and also that TFV makes contact with the genital mucosa and is diluted when it mixes with secretions, we performed dose response experiments to examine the impact of lower concentrations on EM epithelial cells wound closure. As seen in Fig. 1e, incubation of EM epithelial cells with TFV at concentrations ranging from 1 to 100 μg/ml had no effect on TER (1e) or wound closure (not shown) at 24 and 48 h post scratch. Of those concentrations tested, only TFV at 1 mg/ml (3277 μM) interfered with TER recovery and wound closure relative to controls. Based on the findings of others that topical microbicide trials with TFV result in vaginal concentrations close to 1 mg/ml^14^, our findings suggest that wound healing at lower concentrations, assuming these doses are effective in preventing HIV acquisition, would not interfere with trauma-related wound healing. Of note, 1 μg/ml was the threshold for HIV protection in CAPRISA 004^26^.

### Tenofovir Alafenamide (TAF) at clinical oral doses does not affect wound healing by epithelial cells from the FRT

**Figure 2:**
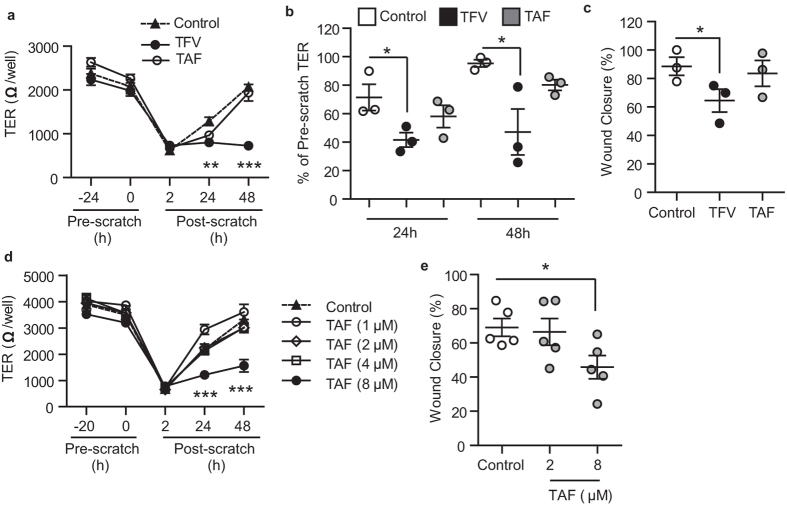
Influence of TFV and TAF on wound healing and recovery of barrier protection by epithelial cells from the uterus. (**a**) TER measurements from polarized uterine epithelial cells incubated with TFV (1 mg/ml; 3277 μM; black circles) or TAF (1 μg/ml; 2 μM; white circles) for 24 h prior to scratch and at 2 h, 24 h and 48 h after scratch. (**b**) Data normalized to % of Pre-scratch TER values from 3 patients at 24 h and 48 h after scratch. (**c**) % of wound closure (Scratch width measurements) from 3 patients at 24 h after scratch. Each circle represents a different patient. The mean and SEM were shown. *p < 0.05. **p < 0.01. ***p < 0.001. (**d**) Effect of increasing concentrations of TAF (1 μM up to 8 μM) on uterine epithelial cell TER. Representative of three independent experiments with 3 different donors. The mean and SEM are shown from triplicate cultures. ***p < 0.001. (**e**) % of wound closure at 24 h after scratch of epithelial cells treated with TAF (2 μM or 8 μM). Each dot represents a different patient (N = 5). *p < 0.05.

Using the same protocols described above for TFV, epithelial cells from the EM were pretreated (24 h) with a clinically relevant concentration of TAF (1 μg/ml, 2 μM), as determined in plasma after oral administration^27^, prior to scratch trauma and then monitored for wound healing. Plasma concentrations were chosen because TAF has only been tested orally and mucosal concentrations of TAF in the FRT are under investigation. As seen in Fig. 2, side by side comparison of TFV (1 mg/ml, 3277 μM) and TAF (1 μg/ml, 2 μM) showed no effect of TAF on TER (2a and 2b) and wound closure at 24 h (2 c), in contrast to TFV inhibition of epithelial cell recovery.

Recognizing that TAF may be used topically in future trials, we examined the effect of increasing concentrations of TAF on wound healing. As shown in Fig. 2d and e, when polarized EM epithelial cells were treated with TAF concentrations ranging from 2 to 8 μM, only the highest concentration (4 μg/ml, 8 μM; 4-fold higher than the plasma levels reached after oral administration) significantly interfered with wound closure and recovery of TER. These findings suggest that concentrations greater than those used orally might compromise wound healing in the FRT.

### TFV, but not TAF, impacts wound healing of epithelial cells from the endocervix and ectocervix

Recognizing that genital injury due to sexual intercourse would mainly affect the lower FRT rather than the endometrium, and that topical TFV administration would reach maximal concentrations in the lower tract, we next determined whether TFV has a similar effect on epithelial cells from the endometrium (EM), endocervix (CX) and ectocervix (ECX). Endocervix was also included in this analysis to model cervical ectopy, a common condition in women during adolescence, pregnancy and contraceptive use that is prone to injury and bleeding^7^.

**Figure 3:**
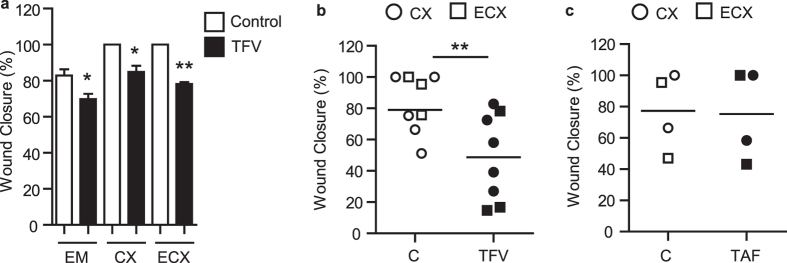
Effect of TFV on EM, CX and ECX epithelial cell wound healing following scratch. (**a**) Confluent cultures of epithelial cells from EM, CX and ECX from the same patient were incubated with TFV (1 mg/ml; 3277 μM) for 24 h prior to scratch with measurements taken at 24 h after scratch. The bars represent mean and SEM from triplicate cultures. *p < 0.05. **p < 0.01. (**b**) Decreased wound closure 24 h after scratch of TFV-treated (1 mg/ml; 3277 μM) epithelial cells from CX (n = 5; circles) and ECX (n = 3; squares). (**c**) Lack of an effect of TAF (1 μg/ml; 2 μM) on endocervical (n = 2; circles) and ectocervical (n = 2; squares) epithelial cells. Each symbol represents a different patient. Mean is shown. **p < 0.01.

Match comparisons of the 3 sites were performed by isolating epithelial cells from EM, CX and ECX from the same patient (Fig. 3a). Epithelial cells from these three anatomical compartments were grown to confluence on cell inserts, treated with TFV (1 mg/ml; 3277 μM) for 24 h, scratched and measured for wound closure at 24 h as described above. As shown in Fig. 3a, incubation with TFV inhibited wound closure of FRT epithelial cells relative to control cells, irrespective of their site of origin. Wound healing delay was confirmed in endocervical tissues from 5 different women and ectocervical tissues from 3 women (Fig. 3b and supplementary movie 4).

We also evaluated the effect of TAF on wound healing by epithelial cells from CX and ECX. As seen in Fig. 3c, treatment with TAF (1 μg/ml, 2 μM) for 24 h prior to scratch had not effect on wound closure of CX (N = 2) or ECX (N = 2) epithelial cells.

As a part of these studies, we found that TER recovery by CX epithelial cells, which are known to form tight junctions, failed to return to control values at 48 h in the presence of TFV. Similar to that seen with EM epithelial cells (Fig. 2a), TAF treatment had no effect on CX epithelial cell TER recovery (not shown).

### Dose dependent inhibition of stromal fibroblast wound closure by TFV and TAF

**Figure 4:**
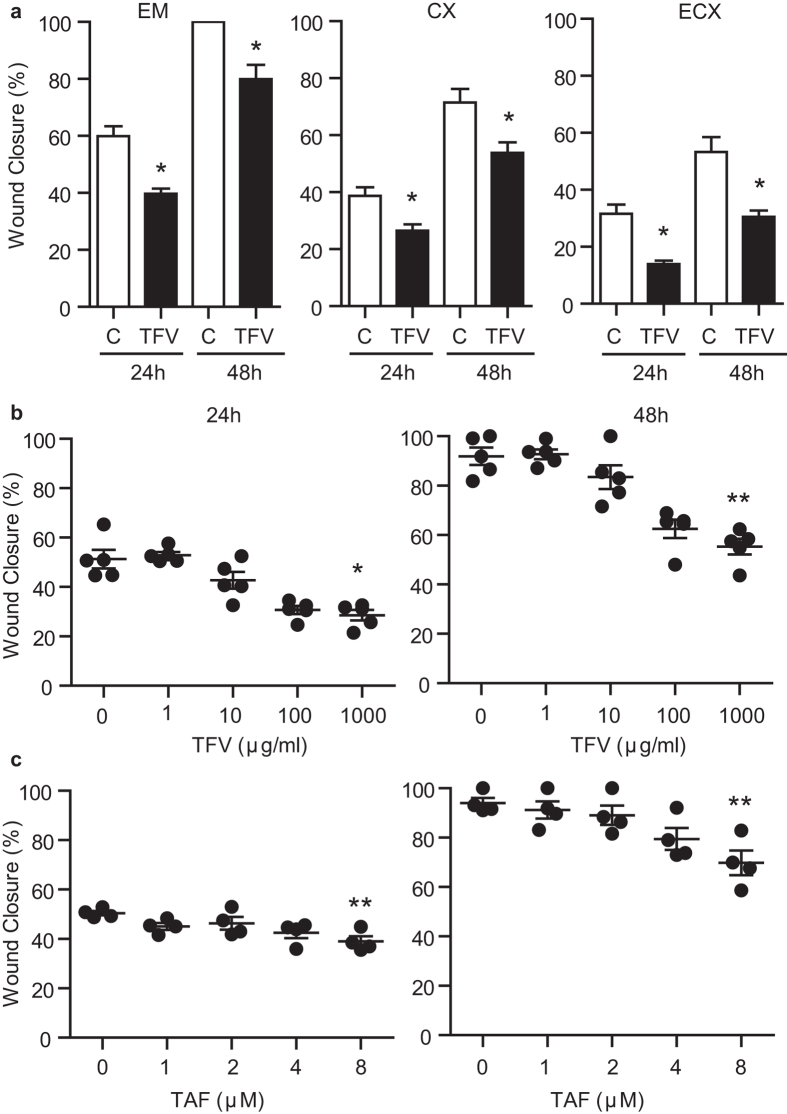
Effect of TFV and TAF on fibroblast wound healing. (**a**) Scratch width measurements were taken to assess wound closure using fibroblasts from EM, CX and ECX from the same patient. Fibroblasts were incubated with TFV (1 mg/ml; 3277 μM) for 24 h before scratch and 24 h and 48 h after scratch. % of wound closure is shown at 24 h and 48 h. The bars represent mean and SEM from triplicate cultures. *p < 0.05. (**b**) Dose response of TFV and TAF (**c**) inhibition of uterine fibroblasts wound healing following scratch. Scratch width measurements were determined for EM fibroblasts incubated with increasing concentrations of TFV (**b**) or TAF (**c**) for 24 h before scratch and 24 h and 48 h after scratch. Each circle represents a different patient (n = 5 for TVF and n = 4 for TAF). The mean and SEM were shown. *p < 0.05. **p < 0.01.

Since genital trauma often breaches the epithelial lining and extends into the underlying tissue, we explored whether TFV might also impact the healing process of stromal fibroblasts. Matched fibroblasts were isolated from different FRT sites (EM, CX and ECX) from the same patient, grown to confluence, and treated with TFV (1 mg/ml; 3277 μM) for 24 h prior to scratch. As shown in Fig. 4a, TFV treatment inhibited wound healing of fibroblasts from the EM, CX and ECX relative to untreated control cells at 24 and 48 h.

To more fully define the responsiveness of fibroblasts to TFV, dose response studies were undertaken using EM fibroblasts. We found that TFV at 1 mg/ml (3277 μM) significantly inhibited wound closure at 24 and 48 h post scratch (Fig. 4b). There was also a trend for 100 μg/ml (327.7 μM) of TFV to inhibit wound healing (p = 0.06).

To determine whether TAF interferes with wound healing of stromal fibroblasts, dose response experiments were carried out in which cells were incubated with increasing concentrations of TAF (1–8 μM; 0.5–4 μg/ml) for 24 h prior to scratch. As seen in Fig. 4c (24 and 48 h), TAF at 1–4 μM (0.5–2 μg/ml) had no effect on fibroblast wound healing. Significant inhibition of closure was observed at 8 μM, which is the same concentration at which impairment of epithelial cell closure following injury was observed (Fig. 3d).

### Comparable intracellular TFV-DP levels result in equal inhibition of barrier function but differential wound closure by TFV and TAF

Recognizing that the doses used in our study to compare TFV and TAF were very different, we performed additional experiments to compare equal doses of both drugs to accurately assess their safety. Clinically, TAF is administered at lower doses than TFV, because intracellular conversion of TAF into TFV-DP is much more efficient than TFV^16^. Therefore, the same doses of these two drugs cannot be directly compared. Thus, to properly compare both drugs, we selected doses that provided equal amounts of intracellular TFV-DP.

**Figure 5:**
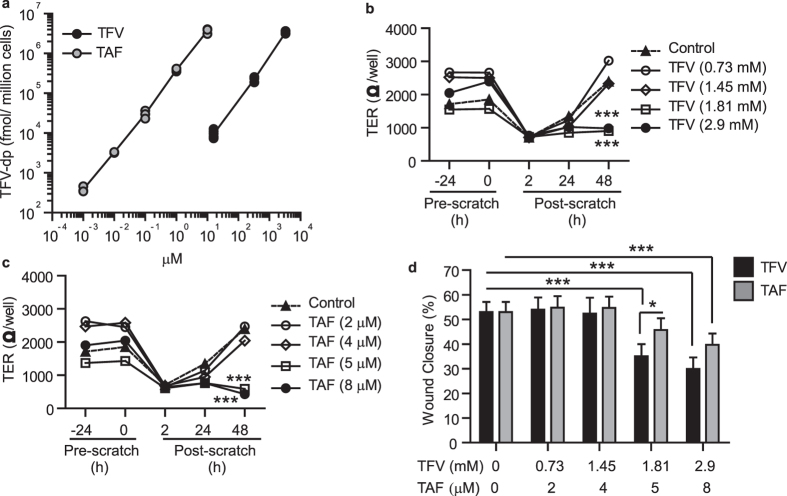
Comparison of intracellular TFV-DP levels in epithelial cells to evaluate TFV and TAF effects on barrier function and wound closure. (**a**) Measurement of intracellular TFV-DP levels in EM epithelial cells following incubation with different doses of TFV (black dots, minimum of n = 3 for each time point) or TAF (grey dots, n = 3 for each time point) for 24 h. (**b** and **c**) TER measurement of EM epithelial cells treated with selected doses of TFV (**b**) or TAF (**c**) that match intracellular concentrations of TFV-DP. Representative of 3 experiments with different donors. Dots represent the mean from triplicate cultures. ***p < 0.001. (**d**) % wound closure after treatment with matched doses of TFV (black bars) and TAF (grey bars). Bars represent mean ± SEM from 4 independent experiments with different donors. *p < 0.05. ***p < 0.001.

First, we assessed intracellular concentrations of TFV-DP in genital epithelial cells following TFV and TAF treatment. EM epithelial cells were treated with different doses of TFV or TAF for 24 h and intracellular levels TFV-DP were determined by liquid chromatography with tandem mass spectrometry (LC-MS/MS) as described before^20^,^23^. As seen in Fig. 5a, TAF was more efficiently converted into TFV-DP than TFV, with lower drug doses resulting in equal levels of intracellular TFV-DP (36-fold difference). Using this data as a standard curve, we then calculated TFV and TAF doses that would result in equal amounts of intracellular TFV-DP to compare both drugs side by side (Supplementary Table 1). EM epithelial cells were then treated with these selected doses for 24 h after which barrier function and wound closure were evaluated. As seen in Fig. 5b (TFV) and 5 c (TAF), regardless of the drug used, equal concentrations of intracellular TFV-DP resulted in equal impairment of TER recovery following scratch. For example, as determined in Fig. 5a, TAF = 5 μM and TFV = 1.8 mM would be expected to have intracellular concentrations of TFV-DP that for each are approximately 1.8 × 10^6^ fmol/million cells. Unexpectedly, as seen in Fig. 5d, wound closure was delayed to a greater extent with TFV than with TAF (TAF = 5 uM and TFV = 1.8 mM). These data suggest that not only intracellular TFV-DP but other additional factors may be responsible for the impairment in wound healing.

Overall, these findings reinforce the importance of ARV dosage and provides evidence that when TAF is considered for topical use, the amount delivered will be important given the possibility of toxic effects on epithelial cells at higher doses.

### TFV but not TAF modifies the acute response of damaged epithelial cells from the FRT

**Figure 6:**
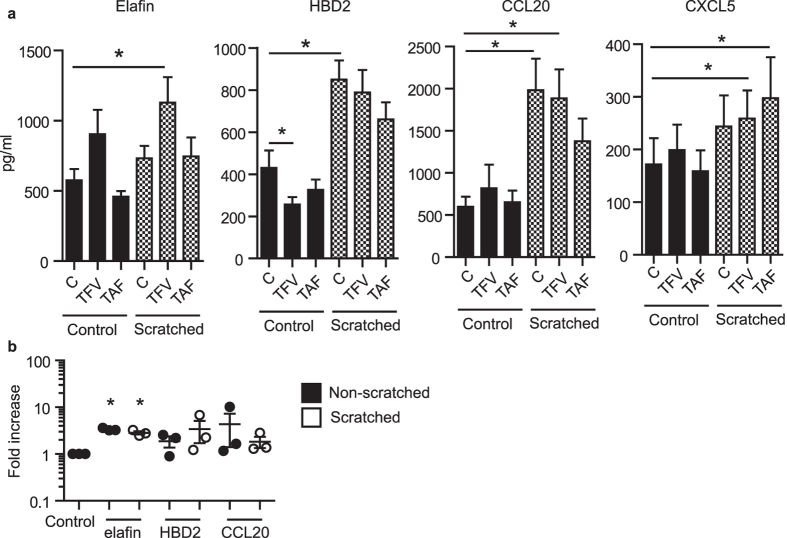
Effect of TFV and TAF on acute secretion of chemokines and antimicrobials by polarized epithelial cells. (**a**) EM epithelial cells were treated with TFV (1 mg/ml; 3277 μM) or TAF (1 μg/ml; 2 μM), scratched and supernatants collected 3 h later and analyzed for the presence of elafin, HBD2, CCL20 and CXCL5. C: untreated control. Bars represent mean ± SEM from 3 independent experiments with 3 different donors run in triplicates. *p < 0.05. (**b**) EM epithelial cells treated with low dose of TFV (0.22 mg/ml), scratched and supernatants collected after 3 h. Graph shows fold increase in elafin, HBD2 and CCL20 compared to control (N = 3). *p < 0.05.

Recognizing that wound healing is a multistep process^28^, we asked if acute responses to injury would be affected by TFV or TAF. Secretions from polarized endometrial epithelial cells were collected 3 h after scratch and analyzed for a combination of chemokines and antimicrobials: elafin, HBD2, MIP-3α (CCL20) and ENA-78 (CXCL5). These molecules were selected due to the roles they play in the acute wound healing and in innate immune protection^28^,^29^,^30^. As seen in Fig. 6a, each of the molecules were measurable in the apical compartment, with no detection in the basal compartment at 3 h post-scratch. When epithelial cells were incubated with TFV (1 mg/ml; 3277 μM) prior to scratch (Fig. 6a, black bars), HBD2 was significantly inhibited by TFV relative to controls, whereas there was a trend towards increased elafin secretion (P = 0.07) with no effect measured for CCL20 and CXCL5. In contrast, TAF (1 μg/ml; 2 μM) had no effect on any of the molecules analyzed. Following injury, HBD2 and CCL20, but not elafin or CXCL5, increased in response to epithelial cell scratch (Fig. 6, hatched bars). TFV pretreatment followed by scratch increased the secretion of elafin, CCL20 and CXCL5 relative to scratch controls. In contrast, TAF treatment only had a significant effect on the secretion of CXCL5, whereas other molecules were unaffected.

Since our data indicates that lower doses of TFV did not delay wound healing, we further investigated whether these doses had an effect on the secretion of antimicrobials. To evaluate low dose TFV, we selected 0.22 mg/ml (0.73 mM) of TFV, the dose that matches intracellular TFV-DP levels obtained after treatment with TAF (1 μg/ml; 2 μM) (Fig. 5). We found that this dose of TFV (0.22 mg/ml; 0.73 mM) increased elafin production before and after scratch, but did not significantly modify HBD2, CCL20 (Fig. 6b) or ENA-78 (not shown). Given that TAF at this intracellular concentration had no effect on cytokine secretion, these findings indicate that TFV and TAF have separate and discrete effects even when intracellular levels of TFV-DP are the same.

### Cell proliferation is not responsible for wound closure

To determine whether epithelial cell and stromal fibroblast closure following injury involves cell proliferation, polarized epithelial cells and confluent fibroblasts were evaluated for changes in proliferation using the CellTiter Aqueous One cell proliferation assay. Relative to matched non-scratch controls, no changes in cell proliferation were detectable in response to trauma at 5 min, 6 h and 24 h post scratch (Supplementary Fig.1). Moreover, when proliferation was measured in the presence of TFV and TAF treated cells, proliferation was not affected (not shown). Visualization of the wound closure process with EM and ECX epithelial cells also suggests that closure is due to cell migration (Supplementary movies 1 and 4). Overall, these findings indicate that closure of epithelial cell wounds from the FRT are most likely due to rapid changes in cell migration rather than proliferation.

## Discussion

In this study we demonstrate that high doses of TFV, equivalent to those used topically in HIV prevention trials, delay wound healing in response to mechanical injury of primary epithelial cells and fibroblasts from the human female reproductive tract (endometrium, cervix and ectocervix). In contrast, TAF, the new prodrug form of TFV, had no effect on wound healing at clinical relevant doses. To the best of our knowledge, our study is the first to report the deleterious impact of TFV on wound healing of human primary epithelial cells and underlying fibroblasts from the upper and lower FRT. Potential implications of wound healing delay for HIV acquisition deserve further investigation.

In the present study, we developed an *in vitro* model to replicate the wound healing process in the human FRT using primary epithelial cells and fibroblasts from the endometrium, endocervix and ectocervix. Studies with human primary cells are important, since cell lines, while informative, do not fully reproduce the characteristics of primary cells^31^,^32^. To the best of our knowledge, ours is the first study using primary cells from the human FRT to demonstrate that TFV interferes with closure of genital wounds. This effect was observed with epithelial cells and fibroblasts from both the upper and lower tract. Based on these findings, we speculate that TFV may impair the healing of both superficial wounds, in which only the epithelium is compromised, and deeper wounds where epithelium and underlying stroma are disrupted. This suggests that in women TFV treatment would delay the healing of genital injuries induced during sex, extending the possible interaction time between HIV and sub-epithelial target cells.

Our studies indicate that beyond wound closure, TFV interferes with the re-establishment of epithelial cell barrier function, measured as TER, both in the EM and CX. Reduced TER is known to correlate with loss of tight junction protection^33^. As demonstrated previously^33^, reduced TER creates a portal of entry for HIV and bacteria into the FRT tissues. Initiated by TNFα in response to HIV, HIV and bacteria remain infectious as they cross the epithelial barrier^33^. In other studies, we have previously shown that TFV induces TNFα secretion by FRT epithelial cells^21^, which may potentially contribute to the failure in re-establishing tight junction barrier protection despite wound closure observed in the present study. Our findings suggest that in the presence of TFV, despite the achievement of wound closure, the protection of the epithelium in providing a selective tight barrier remains compromised. Thus, without measuring epithelial cell barrier function, closure of genital wounds alone may give a false measurement of restored protection.

Given recent evidence that the entire FRT is susceptible to HIV infection, from vagina to ovary^34^, loss of barrier function by uterine epithelial cells might contribute to the risk of HIV infection in the upper tract. Previous data demonstrated that vaginal application of Nonoxynol-9 and universal placebo gel resulted in transcriptional up-regulation of inflammatory mediators in the ectocervix, endocervical canal and endometrium^35^. Taking into account the high doses of TFV applied vaginally (40 mg total at a concentration of 10 mg/ml)^14^ in HIV prevention trials, it is possible that TFV reaches the epithelial cells of the uterus in high enough concentrations to interfere with wound healing and/or inflammatory responses. In this context, although vaginal sex would not be likely to disrupt the endometrial epithelium, epithelial cell barrier protection after disruption due to physiological or pathological causes (i.e. menstruation, ulcers due to ascending infections and endometriosis) may be compromised and increase the possibility of interactions between HIV and target cells.

Another finding in our study is that within 3 h of injury, TFV-treated EM epithelial cells increased the production of several key chemokines/antimicrobials with cell recruitment properties (CCL20, Elafin and CXCL5). In contrast, HBD2, known to have potent anti-HIV activity^36^, was down-regulated by TFV in EM epithelial cells. In previous studies, we have shown induction of CCL20 in TFV-treated ECX epithelial cells^21^, however, more extensive studies are needed to characterized antimicrobial and chemokine production in the ectocervix in response to injury. While some of the molecules increased by TFV in the present study possess anti-HIV activity *in vitro* in the micromolar range (i.e elafin and CCL20)^29^,^30^,^37^, doses in the nanomolar range or lower seem to be preferentially chemotactic^38^,^39^. In particular, CCL20 is chemoattractant for Th17 cells, high susceptible targets for HIV infection^40^,^41^. Our results and those of others^42^ suggest that TFV creates a proinflammatory environment leading to the recruitment of HIV target cells at the wound site. If these findings are confirmed *in vivo*, then it implies that TFV creates an opportunity for HIV to infect at mucosal surfaces by both delaying wound healing and through chemotactic attraction of immune cells susceptible to HIV infection. However, since TFV is very efficient at preventing HIV-infection, further studies are needed to define the balance that exists between inconsistent adherence to TFV use in clinical trials and delayed wound healing, considering the possibility that delayed wound healing and proinflammatory effects of immune cell recruitment might take precedence over the protective antiviral effects of TFV in some cases.

The recognition that TAF is a new TFV pro-drug with higher blood intracellular TFV-DP concentrations, lower plasma exposure (1/10^th^ of the dose) and preferential targeting of lymphoid tissues, suggesting the likelihood of greater antiviral efficacy^17^,^18^,^43^, prompted us to analyze its possible impact on wound healing and chemokine/antimicrobial secretion. In contrast to TFV, TAF had no deleterious effects on wound closure or reestablishment of barrier function. This lack of an effect was observed for both epithelial cells and fibroblasts from upper and lower tract. Remarkably, TAF had little effect on the secretion of chemokines/antimicrobials, with secretion levels indistinguishable from untreated-control cells except for the increase in CXCL5.

Interestingly, the effects or lack thereof of both TFV and TAF were dose-dependent. TFV treatment did not interfere with wound healing at doses lower than those currently used in HIV prevention trials. Lower TFV doses tested that did not delay wound healing (1–100 μg/ml) in the present study, retain their antiretroviral effects *in vitro*^18^ and *in vivo*^26^, and had reduced proinflammatory effects, consistent with previous findings^44^. Meanwhile, TAF delayed wound healing at doses 4-fold higher than those used clinically, providing a wide range for interventions, but cautioning about epithelial toxicity at high doses. These results further reinforce the need for optimization of dosing and administration patterns of ARVs prior to clinical trials. Our findings that TAF does not interfere with wound closure, barrier function or inflammatory responses, adds to the growing body of evidence that TAF may be suitable in the future for pre-exposure prophylaxis in women^19^,^45^. However, TAF has only been used orally for treatment and while oral administration of TAF protected macaques from SHIV acquisition following rectal challenge^46^, other studies indicate that female genial tissue levels of TAF after oral administration in women were low^47^. Further studies are needed to determine correlations between oral TAF and genital tissue drug concentrations and possible topical administration before TAF can be adopted for pre-exposure prophylaxis.

The mechanism by which TFV but not TAF interferes with epithelial and stromal fibroblast wound healing remains to be determined. Wound closure may involve migration, proliferation or a combination of both^48^,^49^. Consistent with previous publications indicating that small wounds close by cell migration and not proliferation^48^, our findings suggest that wound closure and TFV inhibition of wound closure occurs without cell proliferation. Of equal importance is the recognition that both TFV and TAF are converted to TFV-DP inside the cells. Our results demonstrate that comparable intracellular concentrations of TFV-DP from TFV or TAF have the same effects on TER, suggesting that TFV-DP concentrations influence barrier function. However, wound closure was more sensitive to the effects of TFV than TAF, suggesting that the mechanism for delayed wound closure is related to TFV itself in addition to TFV-DP.

Consistent with our findings of TFV impaired wound healing is a recent paper demonstrating that rectal 1% TFV gel application results in marked alterations in epidermal cell proteomics^50^. Proteins affected by TFV included those involved in extracellular matrix, tissue remodeling, epidermal growth and differentiation, tight junctions and cellular stress pathways^50^. Since wound healing of anal injuries is beyond the scope of our present study, future research is needed to investigate if TFV affects wound healing after anal intercourse. To what extent these multiple pathways, all of which are important for wound healing and barrier function, are compromised by TFV in the FRT remains to be determined.

Due to the lack of awareness of the high incidence of genital injuries resulting from consensual and non-consensual sex^51^, HIV prevention trials have not considered the potential impact of genital trauma as an independent risk factor for HIV acquisition. Moreover, evaluation of genital trauma in large clinical trials designed to prevent HIV acquisition is complicated due to numerous factors^52^. For example, superficial wounds induced with sexual intercourse heal within 24–48 h^4^,^53^. Pelvic examinations during CAPRISA 004, were performed quarterly, which would preclude identifying genital injuries related to sexual intercourse and TFV use. Superficial injuries can also be inflicted by tampon use or ARV applicators^54^, adding to the complexity of the evaluation. In addition, non-consensual sex or intimate partner violence is frequently under reported and associated with lower adherence to ARV and medications in general^55^. While genital ulcers and lesions have been described with TFV gel use^54^, the majority of adverse events involving the genitourinary tract observed during clinical trials were mild and did not result in discontinuation of the product^12^,^14^. These would be consistent with our results, and those of others, that TFV in the absence of injury does not alter mucosal barrier integrity. What we demonstrate in the present study is a delay in the wound healing process and an increase in proinflammatory conditions, both of which might result *in situ* in increased opportunities for HIV and target cell interactions. Our findings support the evaluation of genital trauma as a possible risk factor for HIV acquisition in future microbicide trials.

Taken together, the results from this study provide evidence for future clinical studies to investigate the role of ARVs as modifiers of the wound healing process in order to meet the challenges that compromise reproductive health and threaten the lives of women worldwide.

## Additional Information

**How to cite this article**: Rodriguez-Garcia, M. *et al*. Tenofovir Inhibits Wound Healing of Epithelial Cells and Fibroblasts from the Upper and Lower Human Female Reproductive Tract. *Sci. Rep.* **7**, 45725; doi: 10.1038/srep45725 (2017).

**Publisher's note:** Springer Nature remains neutral with regard to jurisdictional claims in published maps and institutional affiliations.

## Supplementary Material

Supplementary Information

Supplementary movie 1

Supplementary movie 2

Supplementary movie 3

Supplementary movie 4
