# Supplementary material for: Tenofovir Inhibits Wound Healing of Epithelial Cells and Fibroblasts from the Upper and Lower Human Female Reproductive Tract
Source: Sci Rep. 2017 Apr 3;7:45725. doi: 10.1038/srep45725 (PMC5377941; doi:10.1038/srep45725)
Supplement: Supplementary Information [file srep45725-s1.pdf]

Tenofovir Inhibits Wound Healing of Epithelial Cells and Fibroblasts from the Upper and Lower Human Female Reproductive Tract

Marta Rodriguez-Garcia, Mickey V. Patel, Zheng Shen, Jack Bodwell, Richard Rossoll,

Charles R. Wira[1].

[1]Department of Microbiology and Immunology, Geisel School of Medicine at Dartmouth, One Medical Center Drive, Lebanon, NH, USA 03756.

*Corresponding author.

Address correspondence to Dr. Marta Rodriguez-Garcia, Department of Microbiology and Immunology, Geisel School of Medicine at Dartmouth, Lebanon, NH 03756. Fax number: 603-650-7717. Telephone number: 603-650-2583.

E-mail address: Marta.Rodriguez.Garcia@dartmouth.edu

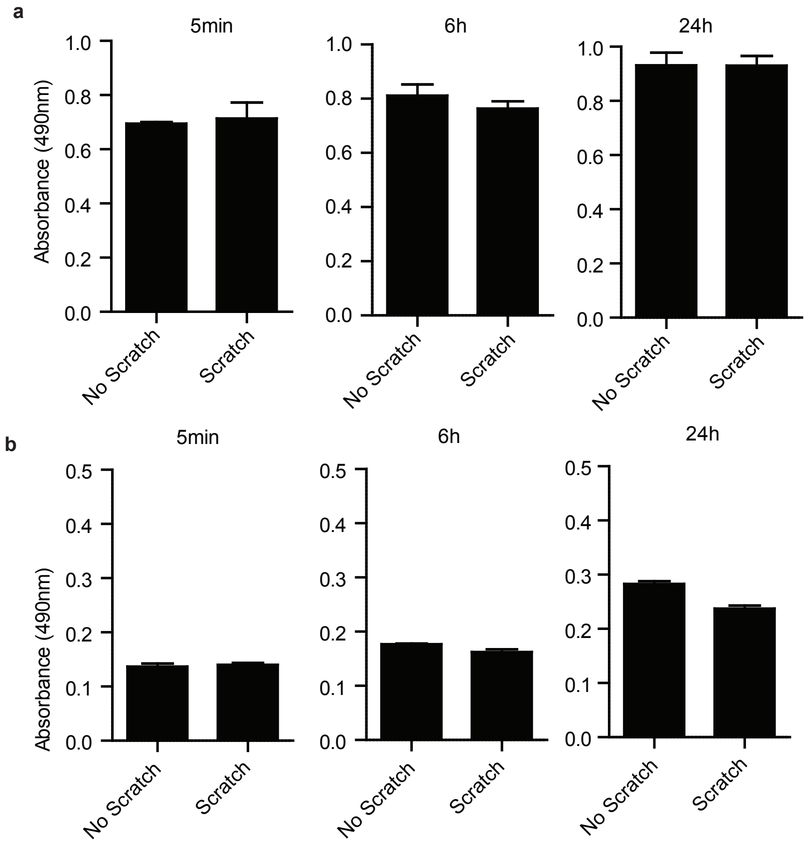

Supp.Fig1. Lack of an effect of wound scratch on uterine epithelial cell and fibroblast proliferation. Epithelial cells and fibroblasts were scratched and analyzed for cell proliferation at 5min, 6h and 24h after scratch using cell proliferation assay. Absorbance at 490 nm was shown in (A) EM epithelial cells and (B) EM fibroblasts. The bars represent mean and SEM from triplicate cultures. Representative of 3 individual donors.

TFV		TAF		Intracellular TFV-DP (fmol/million cell)
**μM**	**mg/ml**	**μM**	**μg/ml**	**$x10^6$**
733.42	0.22	2	1	0.75
1450.84	0.44	4	2	1.48
1809.55	0.55	5	2.5	1.85
2901.985	0.88	8	4	2.97

Supplementary table 1. Intracellular TFV-DP levels in EM epithelial cells after TFV or TAF treatment at different doses.

Supplementary movie 1. Wound closure of endometrial epithelial cells for 48h after scratch.

Supplementary movie 2. Wound closure for a 24h period after scratch of untreated endometrial epithelial cells

Supplementary movie 3. Wound closure for a 24h period after scratch of TFV-treated (1mg/ml) endometrial epithelial cells

Supplementary movie 4. Wound closure of ectocervical epithelial cells for a 24h period after scratch.
